# Editorial: AI-powered musical and entertainment robotics

**DOI:** 10.3389/frobt.2025.1572828

**Published:** 2025-02-25

**Authors:** Huijiang Wang, Josie Hughes, Tetsushi Nonaka, Arsen Abdulali, Thilina Dulantha Lalitharatne, Fumiya Iida

**Affiliations:** ^1^ Bio-Inspired Robotics Lab, Department of Engineering, University of Cambridge, Cambridge, United Kingdom; ^2^ CREATE-Lab, Department of Mechanical Engineering, École Polytechnique Fédérale de Lausanne (EPFL), Lausanne, Switzerland; ^3^ Graduate School of Human Development and Environment, Kobe University, Kobe, Japan; ^4^ School of Engineering and Materials Science, Queen Mary University of London, London, United Kingdom

**Keywords:** human-robot interaction, dexterous manipulation, musical and entertainment robots, machine learning, wearable devices, robotic expressiveness

The convergence of robotics and artificial intelligence (AI) is revolutionizing the field of music and entertainment. Robots are evolving from performing traditional service-oriented tasks to enabling advanced human-robot interaction (HRI) with potential emotional engagement. The pursuit of robotic expressiveness presents new challenges and opportunities in the modeling, design and control of musical and entertainment robots. Current studies mainly work on the design and physical implementation of robots capable of manipulating various musical instruments (Wang et al., 2022; Lim et al., 2012), while the development of socially intelligent robots for real-time HRI remains underexplored. With advancements in AI, robots can now compose and improvise, as well as interpret and respond to human affective states during HRI (McColl et al., 2016; Wang et al., 2024).

**FIGURE 1:**
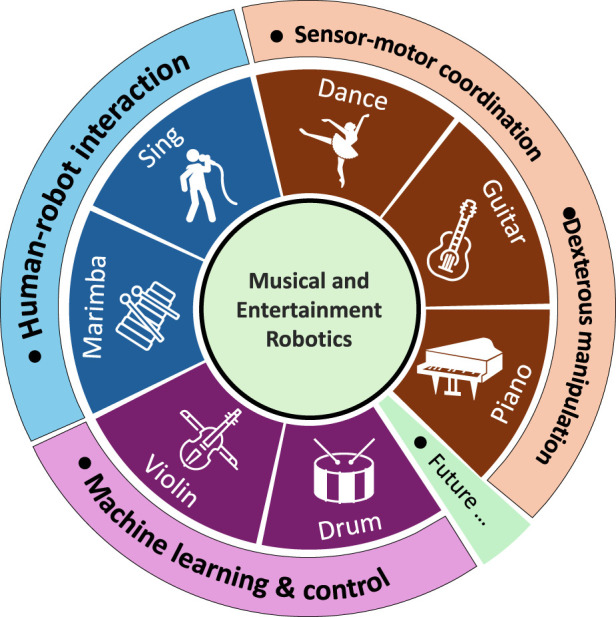
Overview of the musical robots involved in this Research Topic.

This Research Topic was initiated to present the latest developments of AI-powered musical and entertainment robots. As a result of the call, six papers have been accepted and collected in this Research Topic. These articles provide a comprehensive exploration of diverse artistic forms including singing, dancing and musical performance on instruments such as the piano, violin, guitar, drum and marimba. Figure 1 shows an overview of the musical robots investigated in these studies.

Among the contributed works, two articles focused on dexterous manipulation and sensorimotor coordination. Gilday et al. introduced a general-purpose system featuring a parametric hand capable of playing both the piano and performing guitar pick strumming. Unlike existing bespoke robotic musical systems, the proposed hand was designed as a single-piece 3D-printed structure, demonstrating potential for enhanced expressiveness in entertainment applications through the modulation of mechanical properties and actuation modes. The study highlighted that leveraging system-environment interactions enabled diverse, multi-instrument functionalities and variable playing styles with simplified control. Instead of musical instrument playing, Twomey et al. investigated dance performance using wearable soft sensors on the arm to explore whether such devices could enhance artistic expression. Dance movements were modeled as colliders within virtual mass-spring-damper systems, and limb segments were analyzed in local frames to avoid drift issues commonly associated with IMUs. The authors proposed a parallel algorithm to detect improvisational dance movements and control soft wearable actuators which can change size and lighting in response to detected motions. This work exemplified sensorimotor coordination and demonstrated how traditional dance and aesthetics could be enriched by spontaneous wearable-driven movements.

Robot learning and control represent one of the biggest challenges in musical and entertainment robotics, particularly for acquiring manipulation skills and robotic expressiveness. Horigome and Shibuya developed a RL-based controller for a violin-playing robot, a 7-DoF dual-arm system actuated by DC motors. The system mimics human performance with the left arm handling fingering and the right arm controlling bowing movements. The right arm regulates multiple parameters including bowing speed, pressure, sounding point and direction. Analysis of the target sound pressure demonstrated that the robot successfully learned violin-playing techniques and enables expressive performance variations. The robot was automated to play the violin based on musical scores, demonstrating its ability to interpret and execute complex musical tasks. Similarly, Karbasi et al. explored robotic drumming using a two-DoF robotic arm with flexible grippers, which is referred to as ZRob. They employed an RL-based algorithm with a Deep Deterministic Policy Gradient (DDPG) architecture, incorporating both extrinsic and intrinsic reward signals. The results showed that intrinsic rewards triggered the emergence of novel rhythmic patterns. Additionally, the robot’s physical dynamics—embodied intelligence—were found to influence the learning algorithm due to the physical constraints of the drumming setup. This study highlights the interplay between robotic hardware and learning algorithms in achieving expressive musical performance. It can be seen that reinforcement learning continues to be a powerful and widely utilized approach for enabling robots to acquire complex manipulation and expressive skills.

The aforementioned studies have investigated both hardware and software advancements. However, the interaction between these robots and humans has not been explored. Gao et al. investigated synchronization between human musicians and Shimon, a robotic marimba player capable of head and arm movements. Their study revealed that ancillary and social gestures, particularly head movements, significantly enhance temporal synchronization between humans and robots. Through experiments with human participants, the results demonstrated positive social engagement when collaborating with robots in artistic performances. The study also found that social head gestures improved synchronicity slightly more than ancillary or instrumental gestures, providing quantitative insights into the role of non-verbal cues in HRI. Similarly, Nishiyama and Nonaka investigated the concept of “togetherness” in a singing scenario, where human participants coordinated their voices with either another human or a machine (Vocaloid) under non-visual conditions. The study highlighted that human-to-human cooperation achieved higher similarity and anticipatory synchronization compared to human-machine interaction. These findings highlight the critical role of embodiment in enabling natural and effective collaboration, demonstrating how physical presence and human-like traits shape interaction dynamics.

In conclusion, reinforcement learning holds strong potential in tackling the key challenges of equipping musical robots with advanced skills. Current AI-driven robotic systems have demonstrated the feasibility of achieving robotic expressiveness in various musical instruments. However, human-robot interaction presents a more complex Research Topic that requires interdisciplinary collaboration across fields such as robotics, materials science, computer science, psychology, musicology, sociology and ethics.
